# THz Pyro-Optical Detector Based on LiNbO_3_ Whispering Gallery Mode Microdisc Resonator 

**DOI:** 10.3390/s17020258

**Published:** 2017-01-28

**Authors:** Alessandro Cosci, Matteo Cerminara, Gualtiero Nunzi Conti, Silvia Soria, Giancarlo C. Righini, Stefano Pelli

**Affiliations:** 1 Museo Storico della Fisica e Centro Studi e Ricerche “Enrico Fermi”, Piazza del Viminale 1, 00184 Rome, Italy; g.c.righini@ifac.cnr.it; 2 IFAC-CNR, Istituto di Fisica Applicata “Nello Carrara”, Consiglio Nazionale delle Ricerche, Via Madonna del Piano 10, 50019 Sesto Fiorentino, Italy; G.Nunziconti@ifac.cnr.it (G.N.C.); s.soria@ifac.cnr.it (S.S.); s.pelli@ifac.cnr.it (S.P.); 3 Istituto Nazionale di Geofisica e Vulcanologia, Sezione di Pisa, Via della Faggiola 32, 56126 Pisa, Italy; matteo.cerminara@ingv.it

**Keywords:** bolometer, THz, WGM, microdisc, LiNbO_3_

## Abstract

This study analyzes the capabilities of a LiNbO_3_ whispering gallery mode microdisc resonator as a potential bolometer detector in the THz range. The resonator is theoretically characterized in the stationary regime by its thermo-optic and thermal coefficients. Considering a Q-factor of 10^7^, a minimum detectable power of 20 μW was evaluated, three orders of magnitude above its noise equivalent power. This value opens up the feasibility of exploiting LiNbO_3_ disc resonators as sensitive room-temperature detectors in the THz range.

## 1. Introduction

Nowadays THz applications are emerging as a new frontier of technology. Several current studies are concentrating on both generating [1,2,3,4,5] and detecting [6,7,8,9,10] THz radiation. The detection systems mainly involve two different techniques, namely coherent and incoherent [11]. The former uses a heterodyne detection scheme where the signal coming from local oscillator (LO) is mixed with the signal being detected. An important parameter for the mixer devices is the electric field quadratic nonlinearity. Frequently used mixers are superconductor-insulator-superconductor (SIS) tunnel junctions [12], forward biased Schottky barrier diodes (SBDs) [13], superlattices (SLs) [14] and semiconductor and superconducting hot electron bolometers (HEBs) [15]. The main advantage in using heterodyne detection is that both the frequency and phase information are preserved. Where sensitivity is more important than spectral resolution, direct detection can also be used, including devices such as THz antennas [16,17] and thermal detectors, such as Golay cells [18,19], pyroelectric detectors [10] and bolometers [20]. The electro-optic effects induced in LiNbO_3_ and other birefringent crystals are widely studied, and several detectors have already been developed [21,22]. One of the main disadvantages of LiNbO_3_ is its high absorption coefficient in the THz regime, limiting the detection sensitivity. Furthermore, to obtain strong electro-optic effects, applications mainly involve the detection of high peak THz pulses. In this study we propose a new technique for THz sensing by means of a LiNbO_3_ microdisc resonator. Besides its good sensitivity to high peak THz pulses, its small dimensions are extremely sensitive to temperature shifts due to CW (continuous wave) THz absorption as well. Whispering gallery mode (WGM) microresonators are optical resonators characterized by a high quality factor, up to 10^11^ [23], and, therefore, they are suitable for high-precision sensing applications [24]. LiNbO_3_ microdiscs were already widely used for thermal and electro-optic applications [25,26]. Like MgF_2_ [27,28], its birefringent behavior permits extremely fine temperature measurements and stabilization by exploiting the different responses of the two polarization modes, transverse electric (TE) and transverse magnetic (TM) [25,29,30]. The choice of the LiNbO_3_ crystal relies on its higher thermal dependence frequency shift when compared to other crystals such CaF_2_ and MgF_2_ [25,28,31]. The possibility of using WGM resonators such as bolometers was already proposed for the infrared (IR) region by Zhu [32] and Ioppolo [33]. The former study was concentrated on the thermal drift in a toroid silica microresonator promoted by the modulation of a CO_2_ laser. The latter mainly theoretically examined the effect of IR absorption by a liquid-filled hollow WGM sphere resonator due to its thermal expansion. In this work we evaluate the extension of these concepts in the THz domain, proposing an additional application, as pyro-optical detector, to an existent structure [29,30]. In the first part, the thermal response of a LiNbO_3_ microdisc illuminated by THz radiation is theoretically analyzed. The theory takes into account both the thermal expansion and the thermo-optic effect on the resonance shift. Furthermore, the thermal behavior of the device is studied, as well as its characteristic time, when in close contact with a heat sink. The final sensitivity and noise equivalent power are evaluated, pointing out the system limits. 

## 2. Theory

Here, we examine a micro-resonator disc of LiNbO_3_ having a radius *r* = 2 mm and height *h* = 1 mm, placed on an aluminum thermostat. LiNbO_3_ has a density of ρ = 4.648 g/cm^3^ [34], yielding to a mass *m* of about 0.058 g. Its thermodynamic properties are the specific heat C = 0.63 J/(g·K) and the thermal conductivity *k* = 3.92 W/(m·K) [35]. If THz radiation is directed onto the top surface of the microdisc, it will first be largely absorbed by the crystal, and then reflected by the Al thermostat and, finally, further absorbed by the crystal. The radiation will thus experience an effective optical thickness of *2h* = 2 mm. In the THz frequencies above 0.6 THz, the absorption coefficient Α for LiNbO_3_ is above 10 cm^−1^ [36], we can expect that almost 90% of the radiation will be absorbed and converted into heat. In a more specific approach, given an incident power $P_{in}$ we can define the absorbed power:

(1) $$P_{abs}=\left(1-e^{-A\left(\upsilon \right)2h}\right){\left(\frac{n_{LiNbO3}-n_{air}}{n_{LiNbO3}+n_{air}}\right)}^{2}P_{in}=\chi \left(\upsilon \right)\theta \left(\upsilon \right)P_{in}$$

where *χ*(*υ*) is defined as the absorption efficiency, $n_{LiNbO3},n_{air}$ are the refractive indexes of the crystal and of the air, and θ(υ) corresponds to the reflection losses. We define $P_{\theta }=\theta \left(\upsilon \right)\cdot P_{in}$ as the fraction of radiation transmitted inside the crystal after the first reflection. We will see in the following that θ(υ) is of the order of 0.45 for LiNbO_3_. Considering the specific heat of the LiNbO_3_, it is possible to calculate the temperature increase due to the radiation absorption. In order to maximize the thermo-optical response of the device, we consider a z-cut microdisc and a TE mode propagating inside the resonator. Assuming an excitation wavelength at 1500 nm and a TE polarized beam, the thermo-optic coefficient of LiNbO_3_, *α*_to_, is about 3.02 GHz/K, while its drift due to thermal expansion is lower, *α*_te_ ≃ 2.8 GHz/K [25,37], yielding to a total coefficient of *α*_tot_ = *α*_to_ + *α*_te_ = 5.82 GHz/K. Therefore, even a small temperature variation can be detected as a frequency shift. The microresonator limit of detection (LOD) is strictly dependent on the quality factor, and often it is estimated at 1/20 of the resonance width [38]. In the case of a LiNbO_3_ microdisc, it is possible to achieve quality factors (Q-factors) on the order of 10^7^ [29,30], or even of 10^8^ [39], corresponding to a sensitivity of *δν* = 0.1 MHz. Now, under these considerations, it is possible to convert this into a limit of detectable temperature $\Delta T_{lim}$ in which the signal to noise (S/N) ratio is equal to one.

(2) $$\Delta T_{lim}=\frac{\delta \nu }{\alpha_{tot}}=\frac{0.1\;\mathrm{MHz}}{5.82\frac{\mathrm{GHz}}{K}}=17\;\mu K$$

Therefore, the system should be thermally stabilized below 20 μK in order to take advantage of the precision achievable by the microresonator. Otherwise, modulation of the THz source or the signal through a chopper can be used for lock-in detection, as is widely used with Golay cells and pyroelectric detectors. Another solution can involve the measurements of the error signal of the dual-mode temperature stabilization reported for MgF_2_ discs by [27,28], where temperature was stabilized on the order of hundreds of nanokelvin [27,28]. As a first approximation, we consider the system as perfectly insulated, and it is possible to calculate the energy delivered by the THz radiation promoting the minimum detectable shift *δν* of the resonance peak due to the temperature increase $\Delta T_{lim}=\partial v/\alpha$.

(3) $$E=\Delta T_{lim}\cdot C\cdot m=\frac{\partial v}{\alpha }\cdot C\cdot m=0.628\;\mu J$$

where ΔE stands for the energy deposed by the THz radiation, ΔT_res_ is the minimum temperature detectable and C is the thermal capacity. Now, considering that ∆*E* = *P*∙*t*, from Equation (3) it is possible to calculate the THz radiation with power $P_{min}$ versus the irradiating time in order to have a *δν* shift response of the LiNbO_3_ microdisc.

(4) $$P_{min}=\frac{\Delta E}{t\left(s\right)}=\frac{0.628\;\mu J}{t\left(s\right)}$$

**Figure 1:**
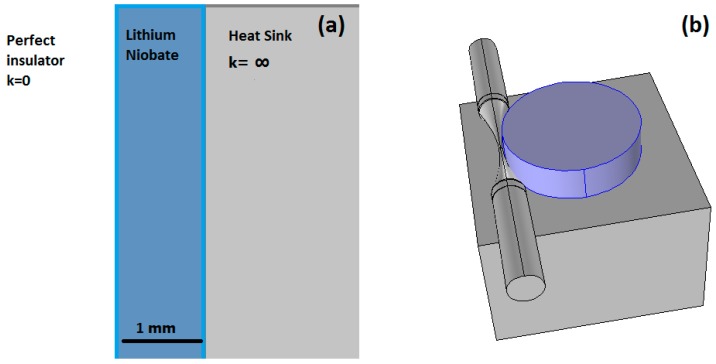
One-dimensional model for theoretical parameter evaluation (**a**). The 1-mm-thick lithium niobate disc has, on one side, a perfect insulator and, on the other one, a perfect heat sink. (**b**) A 3D representation of the LiNbO_3_ microdisc is placed on a metal heat sink and coupled by a tapered fiber.

A system closer to reality has a part of the energy converted into heat that flows into the surrounding system. The easiest configuration is obtained by considering the microdisc leaning on a perfect thermostat, with thermal conductivity *k =* *∞*, surrounded by a perfect insulator, *k* = 0. To simplify the scheme proposed, it is possible to study the problem in 1D, where the whole system can be represented by three layers: the insulator, *h* = 1-mm-thick LiNbO_3_ and the thermostat, as depicted in Figure 1.

In this configuration, using the heat equation, it is possible to calculate the typical time *τ* needed by the system to reach a stationary state when the irradiating thermal power P keeps the sample hotter than the thermostat. The former parameter can be evaluated by the following equation:

(5) $$\tau =\frac{\rho \;h^{2}}{k}C=0.74\;s$$

This equation clearly explains the resistor capacitor circuit (RC) cut-off behavior observed in [32], where the thermal equivalent of the resistivity is dependent on the conductivity k and the heat capacity *C* plays as an electric capacitor. Furthermore, this equation gives the insight for the choice of the resonator dimension and material to optimize the time response. The value of *τ* = 0.74 s indicates the temporal resolution of the device and sets the acquisition rate R at about 1.5 Hz, the frequency corresponding to the typical time of the sample to heat up/cool down of ΔT. 

In order to analyze the detector sensitivity dependence from the THz radiation frequency, we consider the worst scenario where the minimum of the power entering the sample is absorbed: We assume a simple double pass of the THz radiation transmitted inside the resonator $P_{\theta }$, neglecting any additional reflection at the interface with the insulator. We use this absorption profile as a forcing term in the stationary heat equation, obtaining the temperature profile inside the sample: T(0<z<h). Then, we focus on the temperature shift $\Delta T\left(\frac{h}{2}\right)$ in z=h/2, where the tapered fiber is placed. We found that $\Delta T\left(\frac{h}{2}\right)$ is proportional to the flux entering the sample $I_{\theta }=\frac{P_{\theta }}{\pi r^{2}}$, through the dimensional group $\frac{Ih}{k}$:

(6) $$\Delta T\left(\frac{h}{2}\right)=\frac{1}{2}\frac{I_{\theta }h}{k}\cdot \chi \left(\upsilon \right)=\frac{1}{2}\frac{P_{\theta }h}{\pi r^{2}\;k}\cdot \chi \left(\upsilon \right)$$

Using the heat equation, a more rigorous formula for *χ*(*υ*), as a function of the adimensional parameter x=A(υ)·h, could be obtained

(7) $$\chi \left(A\left(\upsilon \right)\cdot h\right)=\chi \left(x\right)=\left(1-e^{-2x}\right)+\frac{2}{x}\left(2e^{-x}-e^{\frac{-x}{2}}-e^{\frac{-3x}{2}}\right)$$

Then, it is possible to evaluate the minimum detectable power $P_{min}$(*υ*) of the incident THz radiation as the incident power $P_{in}$ which promotes a minimum detectable resonance shift $\Delta T_{min}$. This can be easily obtained by inserting the definition of $P_{\theta }$ and Equation (2) in Equation (6),

(8) $$P_{min}\left(\upsilon \right)=\frac{P_{\theta }}{\theta \left(\upsilon \right)}=\Delta T_{min}\frac{2\;\pi r^{2}\cdot k}{h\cdot \chi \left(\upsilon \right)\theta \left(\upsilon \right)}=\frac{\partial v}{\alpha_{tot}}\frac{2\;\pi r^{2}\cdot k}{h\cdot \chi \left(\upsilon \right)\theta \left(\upsilon \right)}$$

Considering that in a complete transmission and absorption of the THz radiation inside the resonator, *χ*(*υ*) = 1 and θ(υ)=1, it is possible to obtain a value for $P_{min}$(*υ*) = 1.69 μW.

**Figure 2:**
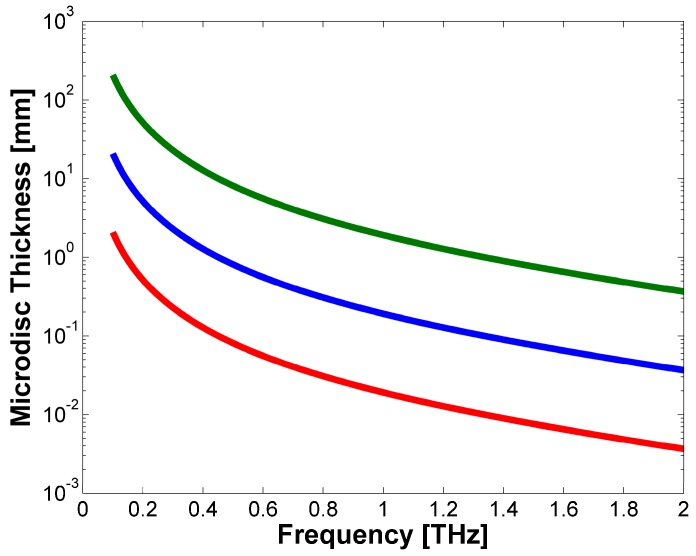
Microdisc resonator thickness needed in order to absorb 10% (red), 50% (blue) and 95% (green) of the radiation versus the THz radiation frequency.

From [36] it is possible to obtain the fit parameter in order to evaluate the values of the crystal absorption A and the refractive index n as a function of the THz radiation frequency υ. From the calculated values it is possible to plot the device thickness needed in order to absorb 10%, 50% and 95% of the incoming THz radiation at different frequencies υ. The obtained values are shown in Figure 2 by means of different colors, red, blue and green, respectively.

**Figure 3:**
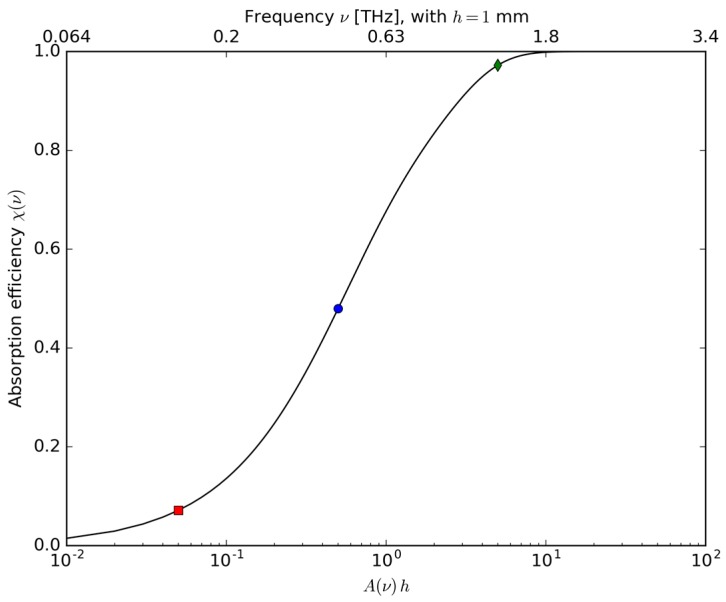
Absorption efficiency χ(ν) versus the optical thickness *x* defined as A(ν)·h, and the values corresponding to an efficiency of 10% (red), 50% (blue) and 95% (green). On the abscissa on the top the dependence of χ(ν) the frequency ν is shown, assuming the value of *h* = 1 mm, as in the case of this article.

As a second result it is possible to show the relationship of the absorption efficiency χ(x) as a function of the optical thickness *x*. Such a dependency is depicted in Figure 3 where, on the top abscissa, instead of the parameter *x*, the dependence from frequency υ is shown, fixing the value of *h* = 1 mm, as in the case proposed here. The red, blue and green points represent the 10%, 50% and 95% absorption efficiencies, respectively. The low absorption limit A≪1/h is on the left of the graph, while the high absorption limit A≫1/h is on the right.

**Figure 4:**
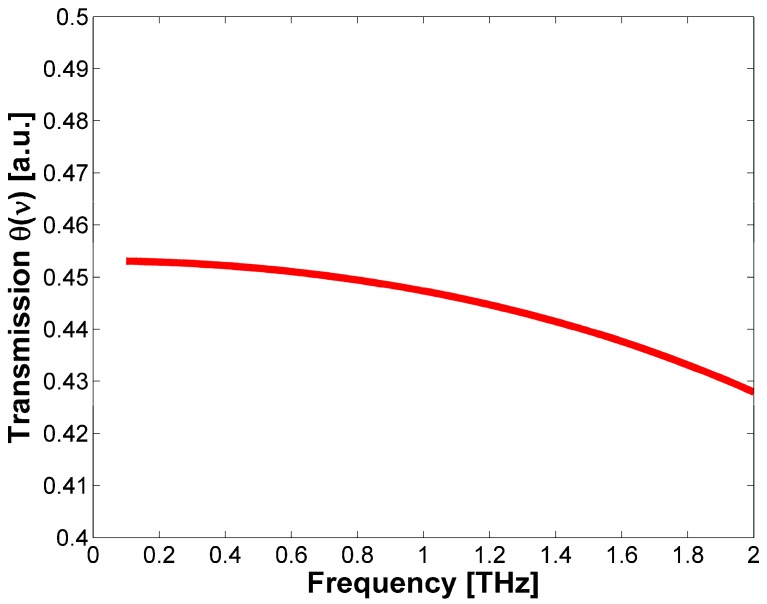
Dependence of THz radiation transmission θ(υ) from the THz radiation frequency υ.

Finally, from the evaluated values of *n*(υ) it is possible to take into account any losses due to the radiation reflection on the device surface. The LiNbO_3_ transmission as a function of the frequency θ(υ) is plotted in Figure 4.

It can be observed that the obtained curve has almost a constant value between 0.45 and 0.43. In the case of high reflectivity and low absorption, the etalon effect could interfere with the measurements. In order to decrease the chances that this phenomenon will occur, different solutions can be taken as a deposition of a thin layer of silica, to match the refractive index, or grinding the resonator surface in the proximity of the heat sink. Furthermore, a simple solution that can give a flat spectral responsivity and a short characteristic time, avoiding etalon effects, could be obtained by placing an absorbing layer on the top of a microresonator disc with a small thickness *h*. Indeed, in this case the absorption is confined to the top of the sample, allowing us to consider the limit x→∞, i.e., *χ* = 1.

To compare a pyro-optical detector with a conventional one based on a thermistor, it is important to also evaluate the noise equivalent power (NEP). Here it is possible to evaluate the thermo-refractive noise by referring to [28], where the case of an MgF_2_ WGM resonator with the same dimensions is considered, and simply inserting the C and *ρ* values for LiNbO_3_ in the following formula: $\langle \partial T_{noise}^{2}\rangle =k_{b}T^{2}/\rho CV$, where V stands for the IR probe beam mode volume. In the case of the disc proposed here, the value obtained for $\langle \partial T_{noise}^{2}\rangle$ is ${\left(456\;nK\right)}^{2}$. This temperature value can then be converted to optical THz power by means of Equation (7). Still, considering *χ*(*υ*) = 1, finally the NEP [40] can be calculated by using Equation (5)

(9) $$NEP=\partial T_{noise}\cdot \frac{2\;\pi r^{2}k}{h}\sqrt{\tau }=2\;\pi r^{2}T\sqrt{\frac{k_{b}\;k}{V}}=9.66\frac{nW}{\sqrt{Hz}}$$

This value is comparable with the one found experimentally in [10] and is well below the one obtained for $P_{min}$. Therefore, it could be assumed that the thermo-refractive noise would not interfere with the measurement, still satisfying the stationary regime. It is, however, necessary to take into account the temperature stabilization needed in order to avoid the temperature fluctuations induced by the surrounding system overwhelming the detector’s precision. 

## 3. Discussion

**Figure 5:**
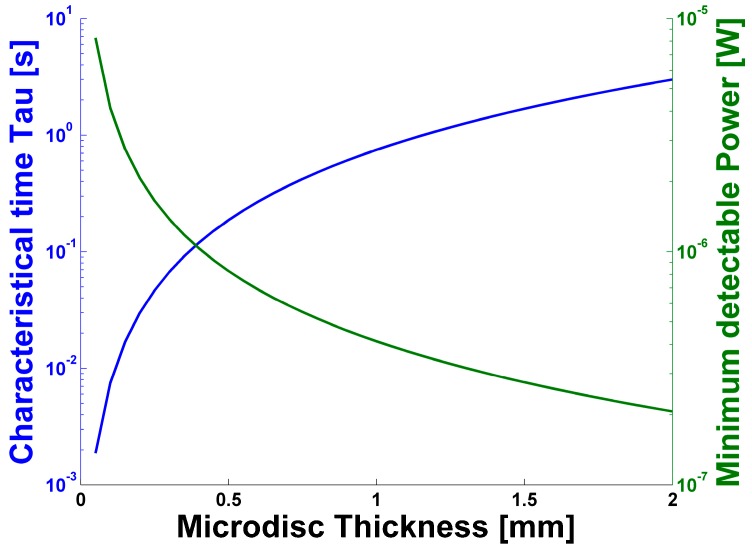
Dependence of the device thickness of τ (blue) and $P_{min}\left(\upsilon \right)$ (green), considering an absorption coefficient Α = 10 cm^−1^ (corresponding to 0.6 THz).

When compared with other conventional bolometers working at room temperature [15,16,20], the system proposed here has a NEP value two orders of magnitude bigger. However, since the proposed sensor is based on the thermo-optic effect, it will not be affected by electromagnetic interference, as could be the case for the pyroelectric sensor. When compared with Golay cells, the solution proposed here offers a higher dynamic range and the possibility to create a rugged structure which can avoid mechanical interference [41]. Furthermore, conventional bolometers have a small size, below 100 μm [15,16,20], in comparison with the 2 mm diameter of the microdisc. Hence, in case of the microresonator device, it is reasonable to expect a further increase in sensitivity, at least by about two orders of magnitude. Another advantage of a sensor based on WGM microresonators relies on the nature of the optical probe, which involves light having a wavelength that suffers low absorption by the crystal and, therefore, does not interfere with the measurements. It also has to be considered that higher precision could be reached with sensors with higher quality factors on the order of 10^9^ [23,27,28]. Regarding the bandwidth, this study mainly concentrates on the feasibility of WGM-based THz bolometers, considering a stationary solution and the minimum power detectable in these conditions. Nevertheless, it is reasonable to expect a working frequency on the order of KHz, as was demonstrated in a similar study involving CO_2_ lasers and a microtoroid resonator [32]. From Equations (5), (6) and (8) it is possible to obtain the complete information about the design of the resonator and the optimal choice of materials. Decreasing the thickness of the microdisc down to 100 μm [42] would increase the acquisition rate two orders of magnitude. However, it is worth noting that decreasing *h* increases the value of $P_{min}$ following Equation (8). Furthermore, a minor optical thickness will yield to lower radiation absorption. Adding these two effects together, in the regime where $h\ll {Α}^{-1}$, the minimum power detectable, $P_{min}$, will be proportional to $1/h^{2}$. Therefore, h cannot be decreased arbitrarily without any impact on $P_{min}$. A plot of Equations (5) and (8) of the dependence of the device thickness of τ and $P_{min}\left(\upsilon \right)$ is shown in Figure 5.

**Figure 6:**
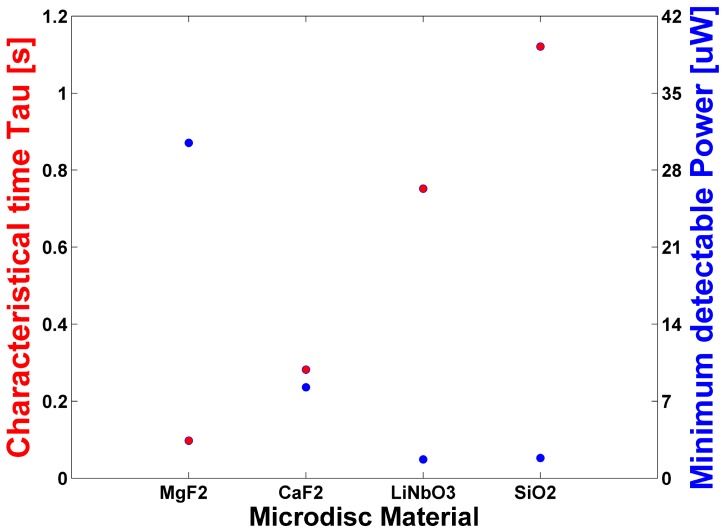
Dependence from the device bulk material of τ (red) and $P_{min}\left(\upsilon \right)$ (blue).

Furthermore, the dependence of *τ* from h found in Equation (5) confirms the choice of the disc geometry instead of the spherical one proposed in [33]. The acquisition rate can be further improved by almost an order of magnitude by using crystals with a lower value of the ratio of *ρ*C/k, as in the case of MgF_2_ or CaF_2_ microdiscs [43]. Moreover, it is possible to observe from Equation (8) that increasing the k value will also increase the NEP. It could be necessary, however, to find a compromise, since higher values of k, even if they help reduce the time constant, also decrease the temperature variation ΔT and, therefore, the system sensitivity. In the system proposed here, LiNbO_3_ was chosen since it provides the highest sensitivity. Furthermore, since LiNbO_3_ is a polar material, pyroelectric charges can be generated on its surface while the temperature changes due to THz radiation absorption [44]. The corresponding electric field can induce a further shift of the resonance and, therefore, enhance the system sensitivity. Nevertheless, a possible drawback can arise from the long lifetime of those charges, which can induce a baseline shift. A comparison of the values of τ and $P_{min}\left(\upsilon \right)$ obtained from microdiscs made of different bulk material, but having the same geometry and dimensions as the one proposed in this work is shown in Figure 6.

When compared to SiO_2_, LiNbO_3_ is more suitable due to its faster response time. Furthermore, the values reported in Figure 6 were obtained considering *χ*(*υ*) = 1, and a proper study involving the efficiency dependence of the wavelength and the crystal type should be done. 

## 4. Conclusions

This study introduced the concept of utilizing LiNbO_3_ microdisc resonators as room-temperature THz detectors. Since the crystal shows a high absorption in the THz range, its thermo-optical features were investigated in order to obtain a THz bolometer. The chosen dimensions and Q-factor (10^8^) were in agreement with standard values obtained in our lab [29,30]. The resonator was considered in ideal experimental conditions in which one side faced an ideal insulator and the other faced a perfect heat sink. A characteristic time of *τ* = 0.74 s for the stationary regime was found, and an equivalent NEP value of 9.66 nW·Hz^-1/2^ was evaluated by considering the thermo-optical fluctuations.

Under these conditions, a minimum detectable power of 2 μW was calculated. This value could be simply improved by employing a microdisc with a higher quality factor or by changing the resonator geometry in order to distribute the radiation mainly in the light-confined mode volume. A Q-factor of the order of 10^9^ [6] could push the detectable power down to 200 nW.
